# Bioconjugated Thymol-Zinc Oxide Nanocomposite as a Selective and Biocompatible Antibacterial Agent against *Staphylococcus* Species

**DOI:** 10.3390/ijms23126770

**Published:** 2022-06-17

**Authors:** Joonho Shin, Atanu Naskar, Dongjoon Ko, Semi Kim, Kwang-sun Kim

**Affiliations:** 1 Department of Chemistry and Chemistry Institute for Functional Materials, Pusan National University, Busan 46241, Korea; shin.joonho@pusan.ac.kr (J.S.); atanunaskar@pusan.ac.kr (A.N.); 2 Microbiome Convergence Research Center, Korea Research Institute of Bioscience and Biotechnology, Daejeon 34141, Korea; kdj8915@kribb.re.kr (D.K.); semikim@kribb.re.kr (S.K.)

**Keywords:** thymol, *Staphylococcus*, antimicrobial, selectivity, biocompatibility

## Abstract

Owing to the rapid spread of antibiotic resistance among *Staphylococcus* species, effective and low-risk alternatives to antibiotics are being actively searched. Thymol (THO), the most abundant component of the oil extracted from thyme, can be considered as a natural antibacterial alternative. However, the low antibacterial activity and non-selectivity of THO limit its usage as a universal anti-*Staphylococcus* agent. Herein, we report the bioconjugation of THO with ZnO nanoparticle (ZO), which resulted in the TZ nanocomposite (NC), as a potent and selective antibacterial agent against *Staphylococcus* species, particularly *S. epidermidis*. The cell-free supernatant (CFS) of ATCC 25923 cultures was employed for the production of TZ NC. Successful production of TZ NC was confirmed via X-ray diffraction (XRD), Fourier-transform infrared (FT-IR) spectroscopy, and ultraviolet–visible (UV–Vis) studies. TZ NC had selective efficacy against *Staphylococcus* species, with MIC values 2–32-fold lower than THO. The antibacterial mechanisms of TZ NC are proposed to involve membrane rupture, suppression of biofilm formation, and modulation of new cell wall and protein-synthesis-associated cellular pathways. Its biocompatibility against HCT116 cells was also checked. Our findings suggest that the TZ nanocomposite could improve the selectivity and bactericidal activity of THO against target species.

## 1. Introduction

The most prevalent bacterial pathogens discovered in human skin diseases are *Staphylococcus* species [1]. Although antibiotics have proven to be efficient for treating such infections, the rapid increase in resistance among *Staphylococcus* species [2,3] has forced the development of new treatments. Natural products and their components are increasingly being used as a viable treatment strategy against *Staphylococcus* infections as their unique modes of action may outcompete existing antibiotic resistance [4].

One of the most important components of thyme (*Thymus vulgaris*) essential oil is thymol (2-isopropyl-5-methyl phenol; THO) [5]. THO has been demonstrated to have antibacterial activity in several trials [6]. For example, THO has bacteriostatic action against most Gram-positive and Gram-negative bacterial species, including *Salmonella typhimurium*, *Escherichia coli*, and *Salmonella enterica*. The antibacterial mechanisms of THO are proposed to involve the destruction of cell membrane integrity, leakage of intracellular materials to the outside, and ultimately bacterial cell death [7,8,9]. However, the commercial use of THO remains limited owing to its poor water solubility, high volatility, and high light sensitivity [10,11]. Further, the selectivity of THO against *Staphylococcus* species has not been documented. Therefore, a practical strategy that improves the efficacy and selectivity of THO against *Staphylococcus* species is required.

Currently, nanoparticles (NPs) are widely used in many aspects of medicine and almost all may be doped or decorated with medically useful compounds [12]. Combining two or more materials is also suggested to improve the properties of the individual materials [13]. Nanocomposites (NCs) generated by conjugating NPs with antibacterial moieties or natural products have been demonstrated as one of the most effective materials for combating antibiotic resistance [14,15,16,17]. In such materials, NPs were found to improve the permeability of bacterial cell membranes, enabling induced uptake by bacterial cells, increased shelf-life, and reduced cytotoxicity of antibiotics [13]. Among NPs, metal oxide NPs are regarded as potential antibacterial agents as they release metals into bacterial cells, where they interact with nucleic acids, functional groups, and proteins [18]. Owing to its chemical stability, economic efficiency, and wide surface area, ZnO NP (ZO) has been a leading antibacterial agent [19]. Furthermore, the ability of ZO to selectively kill Gram-positive bacteria has been demonstrated [20]. However, the inherent cytotoxicity of ZO cannot guarantee biocompatibility when high doses are required.

Many antibacterial metallic NPs have been produced using different chemical procedures, including bottom-up and top-down [21]. Further, biologically mediated biosynthesis or bioconjugated platforms have been designed to mitigate any potentially dangerous outcomes of conventional procedures [22]. Plant extracts, microbes, fungus, and algae are commonly employed in green processes [23]. Compared to plant extracts, the use of cell-free supernatant (CFS) has garnered remarkable attention as it is easy to obtain, lacks seasonal and geographical limits [24], and may tolerate the harmful free metal ions via biosorption or bioaccumulation processes [25,26]. Thus, using CFS in the production of antibacterial NPs lowers the toxicity of metallic antibacterial NPs. As a consequence, the aim of the study is to synthesize a biocompatible antibacterial nanocomposite containing THO and ZO that may selectively inhibit the growth of *Staphylococcus* species. Furthermore, such nanocomposites can reduce the cytotoxicity of ZO, while increasing the antibacterial activity of THO. In this study, we successfully produced a novel THO-ZO (TZ) NC via the bioconjugation of THO with ZO using CFS from ATCC 25923 to increase the potency and selectivity of THO against *Staphylococcus* species. X-ray diffraction (XRD), Fourier-transform Infrared (FT-IR) spectroscopy, and Ultraviolet–Visible (UV–Vis) studies were used to verify and characterize TZ NC. The antibacterial activity of TZ NC was found to be highly selective to *Staphylococcus* species, with minimum inhibitory concentrations (MICs) 2–32-fold lower than that of THO. TZ NC was also found to be highly potent against type and multidrug-resistant (MDR) *S. epidermidis* strains Membrane rupture, suppression of biofilm formation, and modulation of new cell wall and protein-synthesis-associated cellular pathways were identified as the potential antibacterial mechanisms of TZ NC. Further, TZ NC was found to be biocompatible against HCT116 cells at the MIC levels. Our findings suggest that the bioconjugation of THO with ZO by a simple and environmentally friendly method completely remodels the antibacterial properties of THO in a more selective and bactericidal manner.

## 2. Results and Discussion

### 2.1. Material Properties

#### 2.1.1. Phase Composition

**Figure 1:**
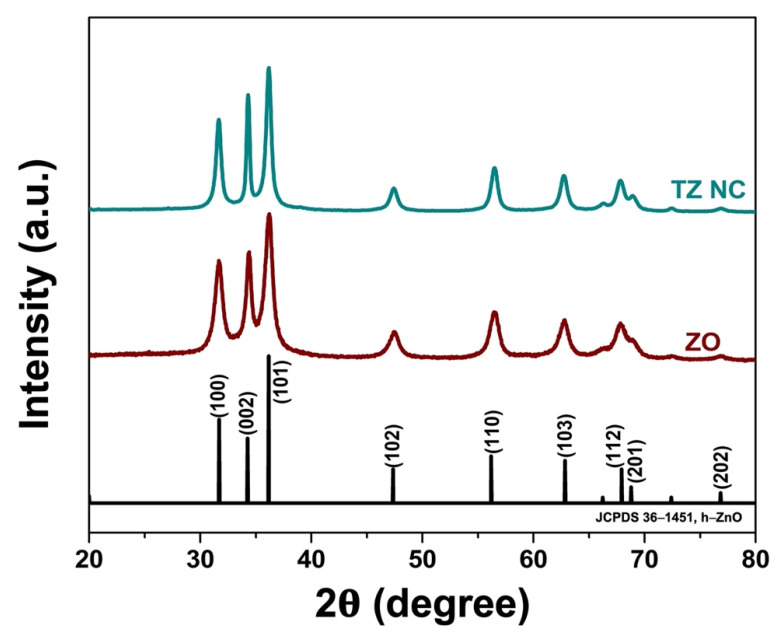
X-ray diffraction (XRD) patterns of the ZO and TZ NC samples.

The crystalline nature of the as synthesized ZnO (ZO) and THO-ZO nanocomposite (TZ NC) samples was analyzed using X-ray diffraction (XRD) analysis. As shown in Figure 1, the XRD reflection peaks of the as-synthesized TZ NC samples are ideally indexed to hexagonal ZO (h-ZO; JCPDS 36–1451). However, there were no characteristic peaks of THO, suggesting that THO did not affect the crystallinity of ZO.

(1) $$D=\frac{K\lambda }{\beta \;cos\;\theta }$$

where *K* is the proportionality constant (*K* = 0.89), *λ* is the X-ray wavelength (0.15406 nm), *β* is the full-width at half maximum for the peak of maximum intensity (in radians), *θ* is the diffraction angle, and *D* is the crystallite size. The *D* values for TZ were approximately 20 nm. Overall, the XRD results confirmed the successful preparation of the ZO samples.

#### 2.1.2. UV–Vis Analysis

**Figure 2:**
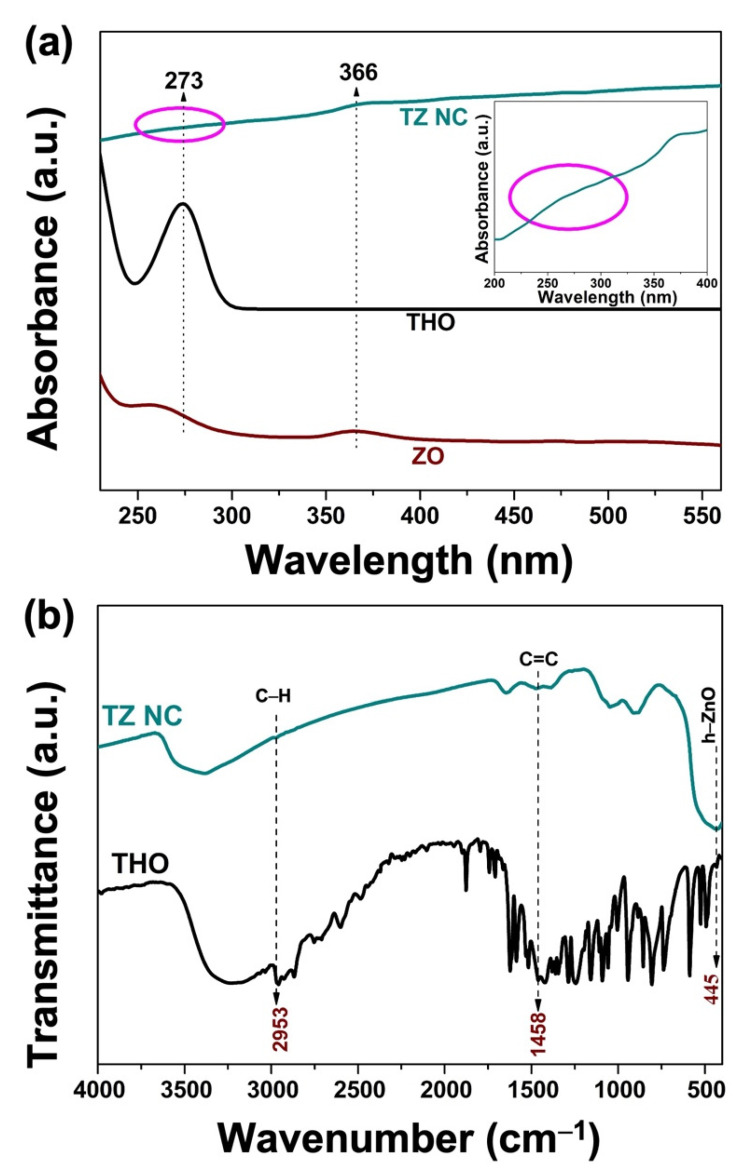
(**a**) UV–vis spectrum of THO, ZO, and TZ NC samples. Inset shows the enlarged spectrum (as marked) of TZ NC sample. (**b**) FT-IR spectrum of THO and TZ NC samples.

To confirm the structural modification and interaction of the thymol skeleton, UV–Vis spectroscopic analysis was performed using the ZO, THO, and TZ NC aqueous suspension dispersions and are shown in Figure 2a. A UV absorption peak near 273 nm was detected in the UV–Vis spectrogram of TZ NC, which could be attributed to the π-π* electron transition overlapped with the vibration of the aromatic ring in THO. Another absorption peak at 366 nm is the characteristic peak of ZO [27]. However, the intensity of the absorption peak is markedly lower than that of naked THO at 273 nm. This phenomenon implied that the THO in TZ NC was more stable than naked THO, thereby requiring more energy to stimulate the π electron transition [28]. This absorption spectrum aligned with the reported absorption spectrum of THO and the THO-reduction derivative commonly produced in aqueous solution and oxygen-loaded conditions. This peak was detected when ZO was conjugated with THO as a distinct and broad shoulder peak at 273 nm (inset of Figure 2a). Therefore, UV–Vis spectrum analysis confirmed the successful formation of TZ NC by the interaction between THO and ZO.

#### 2.1.3. FT-IR Analysis

Infrared spectroscopy was used to characterize the main functional groups of THO and reveal potential interactions between TZ NC and added active phenolic compounds. The FT-IR spectra of pure THO and TZ NC are shown in Figure 2b. In the spectrum of pristine, the characteristic vibrations of THO were observed in the region of 450–4000 cm^−1^, which is ascribed to the hydroxyl and aromatic groups of THO. Further, a broad peak at ~445 cm^−1^ was found to correspond to the stretching vibration of Zn-O of h-ZO. Moreover, peaks corresponding to the main characteristic bonds were found for THO. A weak absorption band at 2953 cm^−1^ was assigned to the C-H vibration in TZ NC [29]. Thus, the vibrations at 1458 cm^−1^ in THO and TZ indicate that C = C stretches in aromatics were conserved after conjugation with ZO [30]. Based on the above analysis, THO and ZO interact in the final TZ NC sample.

### 2.2. Biological Activity

#### Antibacterial Activity

Initially, MIC assays (Table 1) using the microbroth dilution method were performed to evaluate the antibacterial activity of the TZ nanocomposite compared to that of the parental materials (ZO and THO) against representative Gram-negative (*E. coli* ATCC 25922) and Gram-positive (*Staphylococcus aureus* ATCC 25923) type bacterial strains. As shown in Table 1, TZ NC had an 8-fold higher antibacterial activity than ZO and THO against ATCC 25923; however, such increase was not found against the ATCC 25922 strain. This finding suggests that TZ NC is selectively active toward *S. aureus* with better activity compared to THO against *S. aureus*. We proceeded to determine whether such increase in antibacterial activity can be applied against different *Staphylococcus* species, including *S. epidermidis*, which resided with *S. aureus* in human skin, and *S. warneri* [31].

Based on the MIC data (Table 1), the increased antibacterial activity of TZ NC occurred in ATCC 14990 and ATCC 27836 strains, with 2–32-fold different preference to TZ NC. Among the tested strains, *S. epidermidis* was the most preferential target to TZ NC. To validate the preference of *S. epidermidis* to TZ NC, the MIC of TZ NC to an additional type strain of *S. epidermidis* (KCTC 13171) was determined. TZ NC was found to be 16-fold more active than THO against the strain (Table 1). These findings suggest that TZ NC has a preference for *S. epidermidis*. As antibiotic-resistant strains that cannot be controlled using current antibiotics, infections owing to *S. aureus* and *S. epidermidis* have increased, ultimately impacting human health [32,33]. We proceeded to determine whether TZ NC could be used as a potential antibacterial agent against antibiotic resistant strains. Briefly, several methicillin-resistant *S. aureus* (MRSA) and MDR *S. epidermidis* strains were selected. Thereafter, the MIC values of TZ NC against these strains were determined. As shown in Table 1, TZ had better activity than THO in 3 of the 4 MRSA strains (75%), with 2–4-fold decreases in the MIC values, despite a less effective antibacterial activity than that in the type *S. aureus* strain (ATCC 25923). Meanwhile, the efficacy of TZ NC against an MDR *S. epidermidis* (ATCC 12228), was 32-fold, which is the same efficacy against the type strain (ATCC 14990). Therefore, such findings indicate that TZ NC is a potential antibacterial agent that kills *S. epidermidis* species of both type and MDR strains, but is less effective agent against *S. aureus* and its MDR strains.

**Figure 3:**
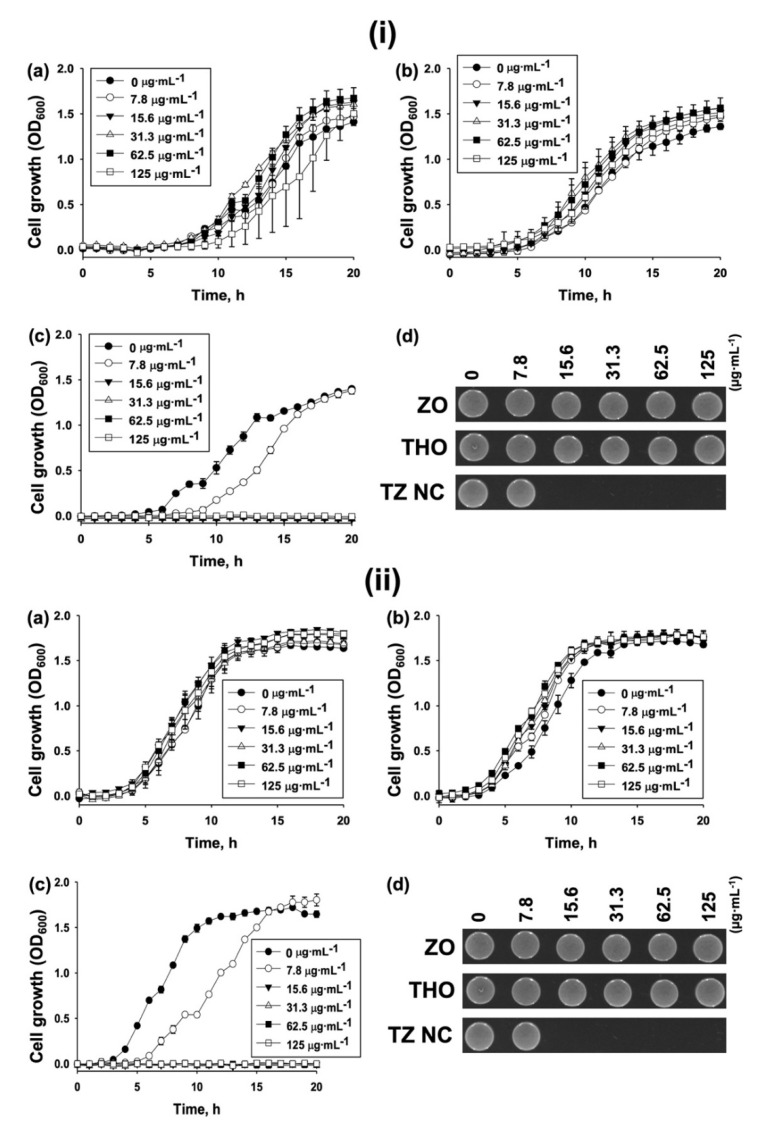
Growth curve analysis. (**i**) Growth of *S. epidermidis* strain (ATCC 14990) at an absorbance of 600 nm (OD_600_) following treatment with different concentrations of (**a**) ZO, (**b**) THO, or (**c**) TZ NC for 20 h. The growth curve data were plotted as average values with standard deviations of *n* = 3 using SigmaPlot (ver. 12.5) (Systat Software Inc., San Jose, CA, USA). (**d**) Cell viability. (**ii**). Growth of MDR *S. epidermidis* strain (ATCC 12228) at OD_600_ following treatment with different concentrations of (**a**) ZO, (**b**) THO, or (**c**) TZ NC for 20 h. The growth curve data were plotted as the average values with standard deviations of *n* = 3 using SigmaPlot (ver. 12.5) (Systat Software Inc., San Jose, CA, USA). (**d**) Cell viability. A fraction of cells from the end point of the growth curves were spotted on LB agar plates and incubated at 37 °C for 24 h. The plate images were captured using ChemiDoc^TM^ MP (Bio-Rad, Hercules, CA, USA) and ImageLab^TM^ Software (ver.5.2.1, Bio-Rad, Hercules, CA, USA). One of the representatives from *n* = 3 is presented.

Growth curves of the *S. epidermidis* strains, including MDR, following treatment with different concentrations (0–125 µg⋅mL^−1^) of THO, ZO, and TZ NC were generated. The growth of *S. epidermidis* (ATCC 14990) following treatment with 125 µg⋅mL^−1^ of ZO and THO was not inhibited (Figure 3(ia,ib)), whereas 15.6 µg⋅mL^−1^ of TZ NC completely inhibited cell growth (Figure 3(ic)), thereby corresponding with the MIC value (Table 1).

A similar result was obtained using THO, ZO, and TZ NC against the MDR *S. epidermidis* (ATCC 12228) strain (Figure 3ii). To differentiate whether bacterial killing by TZ NC is bacteriostatic or bactericidal, additional cell viability assays using the fractions of cultures at the end point of the growth curves were performed. The cells did not grow at the MIC level on LB agar plates (Figure 3(id,iid)). Similarly, additional cell viability assays performed with all strains (Table 1) after 20 h growth confirmed that the MIC values of TZ NC represented the concentrations required for complete bactericidal activity (Figures S1–S3). Collectively, these findings indicate that TZ NC is an efficient bactericidal agent against *S. epidermidis* species.

### 2.3. Plausible Antibacterial Mechanism

#### 2.3.1. Inhibition of Bacterial Biofilm Formation by TZ NC

**Figure 4:**
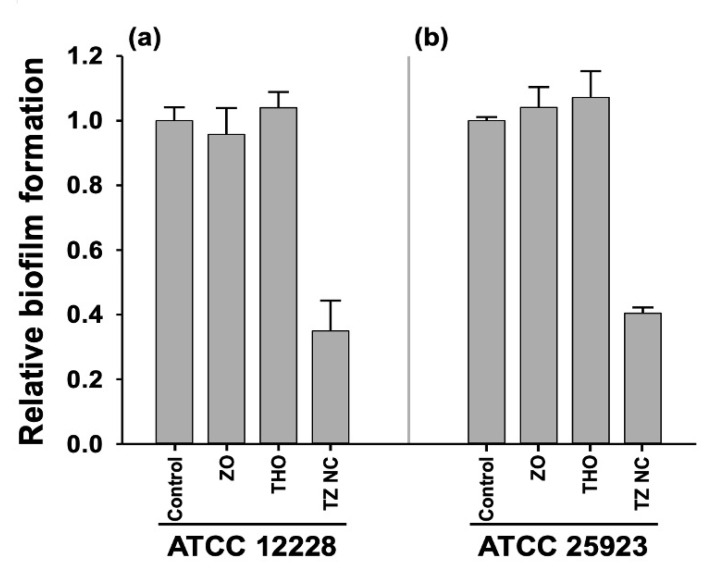
Biofilm formation assays. Relative biofilm formation (OD_595_/OD_600_) was determined for cells of (**a**) MDR *S. epidermidis* (ATCC 12228) and (**b**) type *S. aureus* (ATCC 25923) strains under the presence or absence of ZO, THO, and TZ NC. The values shown in the graphs represent average values with standard deviation of *n* = 10 experiments (*p* < 0.05). The data were analyzed using GraphPad Prism 8 (GraphPad Software Inc., San Diego, CA, USA).

*S. epidermidis* is a nosocomial pathogen owing to its intrinsic ability to form biofilms on the surface of implantable medical devices, which is often accompanied by multidrug resistance [37] and increased antibiotic tolerance [38]. As TZ NC effectively kills *S. epidermidis* strains (Table 1), the inhibition of biofilm formation is expected to be a potential antibacterial mechanism of TZ NC. To verify this hypothesis, CV assays were performed using the MDR *S. epidermidis* (ATCC 12228) strain. At the sub-MIC (3.9 µg·mL^−1^), TZ NC inhibited the biofilm formation of *S. epidermidis* by more than 50%; however, THO and ZO did not inhibit biofilm formation (Figure 4a). To determine whether the inhibition of biofilm formation could explain the higher activity of TZ NCs against *S. epidermidis* compared to *S. aureus*, CV assays were performed against *S. aureus* (ATCC 25923) cells treated with TZ NC. At the sublethal concentration (15.6 µg·mL^−1^), TZ NC effectively reduced *S. aureus* biofilm formation by more than 50% (Figure 4b), suggesting that anti-biofilm activity is one of the antibacterial mechanisms of TZ NC. However, this mechanism cannot explain the high activity of TZ NC against *S. epidermidis*. TZ NC may thus be used to target hyper-biofilm forming strains instead of *Staphylococcus* species; however, this speculation cannot be accepted as TZ NC did not display antibacterial and anti-biofilm formation activities against *Pseudomonas aeruginosa*, a high biofilm-producing Gram-negative strain (data not shown). Therefore, the degree of biofilm is not the determinant of TZ NC action. Instead, other mechanisms are involved in the selective activity of TZ NC against *S. epidermidis* species.

#### 2.3.2. Morphological Characterization of Bacteria: Bursting from Within

**Figure 5:**
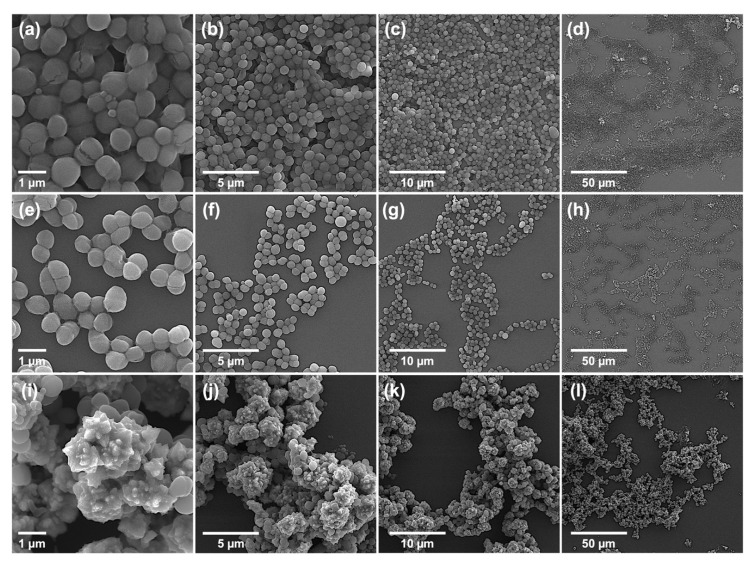
Cell morphology analysis. Scanning electron microscopy (SEM) images of MDR *S. epidermidis* cells of ATCC 12228 without (**a**–**d**) treatment with either 1/4 MIC (**e**–**h**) or 1/2 MIC (**i**–**l**) of TZ NC, respectively were shown. No membrane disruption was shown in 1/4 MIC of TZ NC treated cells (**e**–**h**) compared to non-treated cells. The whole membrane was aggregated with TZ NC in cells treated with 1/2 MIC of TZ NC (**i**–**l**), inducing partial membrane rupture.

It is well known that the antibacterial action of NPs predominantly targets biofilms and cell walls [39], leading to membrane disruption. To determine whether the membrane disruption is the potential action mechanism of TZ NC, the morphologies of type *S. aureus* (ATCC 25923) and MDR *S. epidermidis* (ATCC 12228) cells with or without treatment of TZ NC at the sublethal concentrations (1/2 or 1/4 MIC) were determined using SEM and FE-SEM image analysis. From the results, several morphological features of cells by TZ NC, which was characterized by FE-SEM (Figure S4), were identified. First, *S. aureus* and *S. epidermidis* cells without treating TZ NC were associated together as groups by mucous membrane structures (Figure 5a–d, Figures S5 and S6). However, the distance between cells was increased and grouped cells were separated when 1/4 MIC of TZ NC was applied (Figure 5e–h). This supports the inhibition of biofilm formation phenotype by TZ NC shown in CV assays (Figure 4). Second, cells treated with 1/2 MIC of TZ NC were completely aggregated with TZ NC and formed a larger complex than cells without TZ NC treatment, causing membrane rupture in some cases (Figure 5i–l and Figure S6d). This phenotypic transformation is a unique and undiscovered phenotype by previous NP studies, and analyzing its structure helps to explain how TZ NC enters cell membranes and destroys bacteria. Additional ROS measurement showed that TZ NC did not induce the ROS (Reactive Oxygen Species) production (data not shown), implying that this radical activity kills bacterial cells even before the production of ROS as a result of cell membrane disruption.

#### 2.3.3. Synergistic Action of TZ NC with Antibiotics

In many reports, different drugs were found to exhibit synergistic activity when their mechanisms of action were the same [40]. Therefore, we opted to identify synergistic antibiotics that increased the activities of TZ NC in the killing of *S. epidermidis*. Briefly, a pre-made Sensititre^TM^ Gram Positive MIC plate (Cat. No. GPALL1F), which was coated with different concentrations of commercial antibiotics (Figure S7a), was used to determine the MIC values following treatment with the sub-MIC (7.8 µg·mL^−1^) of TZ NC against ATCC 14990 strain. The MIC of ampicillin (AMP), cefoxitin (FOXS), chloramphenicol (CHL), daptomycin (DAP), erythromycin (ERY), levofloxacin (LEVO), linezolid (LZD), nitrofurantoin (NIT), penicillin (PEN), and tigecycline (TGC) was found to be lowered by TZ NC (Table 2; Figure S7b,c). Further, TZ NC had the strongest effect on LZD, PEN, and TGC, which had 4-fold lower MIC values. Based on the inhibitory pathways of LZD, PEN, and TGC, which are inhibitors of protein synthesis [41], cell wall synthesis [42], and translation [43], respectively, TZ NC might initially damage the cell wall by inducing bursting from within, as shown in Figure 5, and block cellular protein synthesis.

### 2.4. Biocompatibility of TZ NC

**Figure 6:**
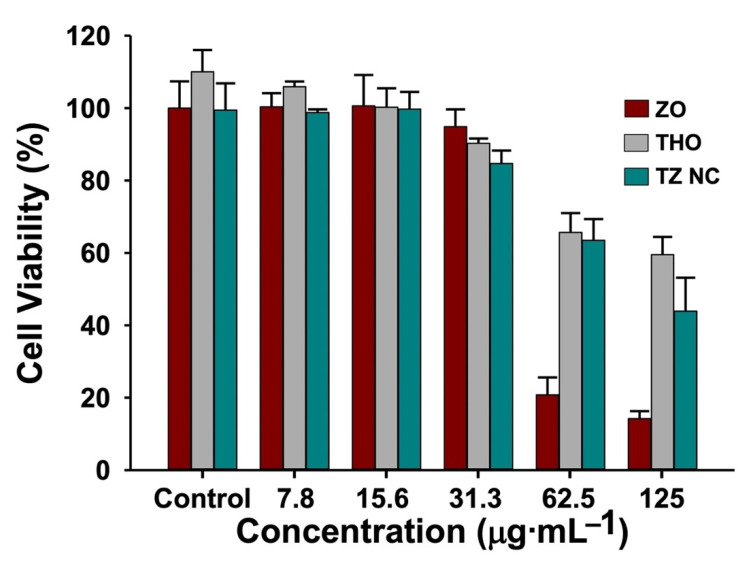
Biocompatibility assays. WST assays were performed to determine the viability of HCT116 cells in the presence of various concentrations of ZO, THO, and TZ NC after 24 h. The values shown in the graphs are averages of *n* = 3 with standard deviation (*p* < 0.05). Data were analyzed using GraphPad Prism 8 (GraphPad Software, Inc., San Diego, CA, USA).

The biocompatibility of TZ NC was evaluated via the WST assay using laboratory available HCT116 cells treated with different concentrations of ZO, THO, and TZ NCs for 24 h. As shown in Figure 6, HCT116 cells were >90% viable following treatment with 31.3 µg·mL^−1^ ZO, THO, and TZ NC. Further, cell viability was >70% after treatment with both TZ NC and THO, and <20% with 62.5–125 µg·mL^−1^ of ZO. As the MIC of TZ NC against *S. epidermidis* was lower than 15.6 µg·mL^−1^, TZ NC could be a potential nano-antibacterial composite against *S. epidermidis* species that retains the biocompatibility of THO.

## 3. Materials and Methods

### 3.1. Biosynthesis of TZ NC

Initially, a 100 mM THO (5-Methyl-2-isopropylphenol, 2-isopropyl-5-methylphenol, 5-Methyl-2-(1-methylethyl) phenol, Sigma-Aldrich, Saint Louis MO, USA) solution in 99% ethanol was prepared as a stock solution. Thereafter, in a separate beaker, 20 mM of zinc nitrate (Cat. No. 96482, Sigma-Aldrich, Saint Louis, MO, USA) aqueous solution was prepared in 100 mL nuclease free water (NFW). A 500 µL aliquot of the THO solution was blended with the zinc nitrate solution via dropwise addition with continuous stirring; the pH of the mixture was adjusted to 11 using aqueous NaOH (2 M) solution. The resulting solution was saturated via incubation at 60 °C for 10 min, followed by stirring for 3 min at 200 rpm. The resulting deep-orange-colored mixtures were then homogenized with 100 mL of CFS from the cultures of *S. aureus* ATCC 25923 (ATCC; American Type Culture Collection; www.atcc.org, Manassas, VA, USA) in Luria–Bertani (LB) broth to reach a stationary phase for overnight stirring at 37 °C and 230 rpm. Of note, the same procedure using CFS from the *E. coli* culture was unsuccessful in the biosynthesis (data not shown). The sample was then collected by centrifugation at 8000 rpm for 10 min at 4 °C and washed twice with 5 mL of NFW and 99% ethanol, respectively. Finally, the fine particles were collected and stored in an airtight container for additional investigation.

### 3.2. Characterization

#### 3.2.1. Properties of the Materials

The XRD patterns of as-prepared ZO and TZ were acquired in the 2θ range of 20–80° using an X-ray diffractometer (D8 Advance with the DAVINCI design, Bruker, MA, USA) equipped with a Ni-filtered Cu Kα radiation source (λ = 1.5406 Å). The FT-IR spectral study was performed using a PerkinElmer Model Spectrum Two (Thermo Electron Corporation, Madison, WI, USA). The UV–Vis spectra were obtained using a UV–Vis spectrophotometer (Lambda 465, PerkinElmer, Waltham, MA, USA).

#### 3.2.2. Preparation of Bacterial Cells

Bacterial cells were purchased from American Type Culture Collection (ATCC; https://www.atcc.org, Manassas, VA, USA) and Korean Collection for Type Cultures (KCTC; https://kctc.kribb.re.kr/, Jeongeup, Korea) and diluted to an optical density of 0.5 McFarland turbidity using a Sensititre^TM^ Nephelometer (Thermo Fisher Scientific, Waltham, MA, USA) to prepare freshly grown bacterial colonies on LB agar plates for the antibacterial activity assays and cell morphology analysis. Individual cell suspensions were inoculated in Sensititre^TM^ Cation adjusted Mueller–Hinton broth (MHB) w/TES (Cat. No. T3462; Thermo Fisher Scientific, Waltham, MA, USA), as described in a previous report [44].

#### 3.2.3. Determination of MICs

The antibacterial activity of the synthesized samples was assessed using 96-well-based MIC determination assays as described in a previous report [45]. Briefly, the bacterial cells with 0.5 McFarland turbidity were 1000-fold diluted in MHB. ZO, THO, and TZ NC samples (5 mg·mL^−1^ for each) were prepared in NFW and serially diluted with NFW to concentrations of 3.9–500 µg·mL^−1^. Thereafter, 5 µL of each diluted sample was inoculated into 45 µL of the targeted bacterial cell suspensions in a 96-well plate (Cat. No. 34296, SPL, Daejeon, Korea). The samples were then incubated with shaking at 500 rpm for 20 h at 37 °C. The defined MIC values, which highlighted the bactericidal action of the samples, were further determined by spotting 5 µL of grown cells on LB agar plates and allowing the cells to grow for 24 h at 37 °C. After incubation, images were obtained using ChemiDoc^TM^ MP (Bio-Rad, Hercules, CA, USA) and ImageLab^TM^ Software (ver.5.2.1, Bio-Rad, Hercules, CA, USA). One of the representatives from *n* = 3 is presented.

#### 3.2.4. Growth Curve Analysis

Bacterial cells were prepared to an optical density of 0.5 McFarland turbidity using a Sensititre^TM^ Nephelometer (Thermo Fisher Scientific, Waltham, MA, USA). Cells diluted 1000-fold in MHB (195 µL) were mixed with or without 5 µL of ZO, THO, or TZ NC at specific concentrations and dispensed into a 96-well microplate (Cat. No. 30096, SPL, Daejeon, Korea). The plate was shaken at 500 rpm for 20 h and growth (absorbance at 600 nm (OD_600_)) was monitored every 1 h using a SPECTROStar^®^ Nano (BMG LABTECH GmbH, Ortenber, Germany). The resulting data were analyzed using MARS V4.01 R2 software (BMG Labtech GmbH, Ortenber, Germany). To monitor the bactericidal activity of the end point of growth, cells with different concentrations of ZO, THO, and TZ NC after 20 h of growth were diluted. Thereafter, 5 µL of the diluted cells was spotted on LB agar plates and incubated for 24 h at 37 °C. Images were obtained using ChemiDoc^TM^ MP (Bio-Rad, Hercules, CA, USA) and ImageLab^TM^ Software (ver. 5.2.1, Bio-Rad, Hercules, CA, USA). One of the representatives from *n* = 3 is presented.

#### 3.2.5. Crystal Violet Assays

To evaluate biofilm formation by bacterial cells, crystal violet (CV) assays were performed as described [46] with the following modifications: ATCC 25923 and ATCC 12228 were grown in LB broth for 20 h at 37 °C and diluted to 5 × 10^6^ CFU·mL^−1^ in a fresh MHB broth with or without one-fourth MIC of ZO, THO, and TZ NC. Relative biofilm formation was calculated by dividing the absorbance values at 595 nm (OD_595_) by those obtained at 600 nm (OD_600_). The relative biofilm formation is expressed as the average values with standard deviations from *n* = 10 experiments.

#### 3.2.6. Morphological Characterization of Bacterial Cells and Nanocomposite

Morphological analysis of cells was performed [47]. Briefly, six samples from MIC plates (Section 3.2.3) not treated or treated with the 1/4 MIC and 1/2 MIC of TZ NC against ATCC 25923 and ATCC 12228 were collected via centrifugation at 10,000× *g* for 1 min. The resulting cell pellets were resuspended in 500 µL of PBS containing 2% formaldehyde and 1% glutaraldehyde, and centrifuged for 5 min. The final cell pellet was washed twice and resuspended in 1 mL of NFW. Ten-microliter aliquots were collected from the suspension and deposited on a silicon wafer (5 × 5 mm, Namkang Hi-Tech Co., Ltd., Seongnam, Korea) for drying at room temperature. VEGA3, a versatile tungsten thermionic emission scanning electron microscopy (SEM) system (TESCAN, Fuveau, France) and FE-SEM, a high-resolution SEM with a field emission gun (FEG) electron source system (JEOL Ltd, JSM 6700F, Tokyo, Japan), was used to image bacterial cells and nanomaterials on the dried wafer according to the manufacturer’s protocol. The morphological characterization of bacterial cells was performed using SEM at magnifications of 2.3, 11.6, 23.2, and 60.0 kx at WD 3.7 to 4.4, and FE-SEM at magnifications of 2.7 and 60.0 kx at WD 6.9 to 8.1. The FE-SEM was used to image nanomaterials at magnifications of 60.0 kx at WD 2.0 to 3.3. 

#### 3.2.7. Screening of Synergistic Antibiotics with TZ NC

To identify synergistic antibiotics with TZ, 0.5 McFarland turbidity ATCC 14990 or ATCC 12228 cell suspension in MHB media (195 μL) with or without 5 μL of TZ (7.8 µg·mL^−1^) was added to a Sensititre^TM^ Gram Positive MIC plate (Cat. No. GPALL1F, Thermo Fisher Scientific, Waltham, MA, USA) as described in a previous report [48]. Thereafter, the MIC values of individual antibiotics were determined. To differentiate between the NP aggregates and grown cells, 0.5 mg·mL^−1^ of 2-(4-Iodophenyl)-3-(4-nitrophenyl)-5-phenyl-2H-tetrazolium chloride (INT) was added to each well to function as a cell growth indicator and the reducing power of living cells was used to quantitatively measure viability according to the manufacturer’s protocol [32]. The resulting images of the 96-well plates for individual samples with the indicated concentrations were captured.

#### 3.2.8. Biocompatibility Assays

The HCT116 cell line from the ATCC was maintained in RPMI1640 with 10% of fetal bovine serum at 37 °C in 5% CO_2_. Cell viability was determined via the colorimetric WST assay (Ez-Cytox; DoGenBio, Seoul, Korea). Cells were seeded in 96-well plates at a density of 5000 cells per well and incubated for 24 h. Thereafter, cells were incubated for 24 h in the presence of ZO and TZ NC samples at concentrations ranging from 7.8 to 125 µg·mL^−1^ in distilled water, and then with the WST reagent (1/10 of the medium volume). The amount of formazan dye formed was determined by measuring the absorbance at 450 nm (OD_450_) using a SPECTROStar^®^ Nano (BMG Labtech GmbH, Ortenber, Germany).

#### 3.2.9. Statistical Analysis

Statistical analyses for the crystal violet assay and biocompatibility assay were performed using GraphPad Prism 8 (GraphPad Software, Inc., San Diego, CA, USA). The biological replicate data are presented as average values with standard deviation. Growth curve data were processed using MARS V4.01 R2 software (BMG Labtech GmbH, Ortenber, Germany) and plotted as average values with standard deviations using SigmaPlot (ver. 12.5) (Systat Software Inc., San Jose, CA, USA).

## 4. Conclusions

Herein, the use of the CFS of *S. aureus* ATCC 25923 to produce TZ NC was identified as an environmentally friendly, cost-effective, and simple technique. TZ NC was identified as the most selective and effective bactericidal agent against *S. epidermidis* strains. Based on the morphology analysis, biofilm formation assays, and synergistic antibiotic screenings, the plausible antibacterial mechanisms of TZ NC may involve disruption of cells with reduced biofilm formation and modulation of cellular pathways associated with new cell wall and protein synthesis. TZ NC was also found to be biocompatible at the MIC level. To our knowledge, this is the first study to demonstrate that the bioconjugation of THO, a natural antibacterial flavonoid compound, with ZO, a Gram-positive bacteria selective NP, fully remodels the antibacterial properties of THO in a more selective and bactericidal manner against *S. epidermidis* and maintains the biocompatibility of THO. Although ZO was only used to modify the function of THO in this study, the materials or the method could be widely used in the remodeling of other low active and non-selective natural antibacterial agents, such as flavonoids.

## Figures and Tables

**Table 1:** Minimum inhibitory concentrations (MICs) of ZO, THO, and TZ NC against Gram-negative *E. coli* and Gram-positive *Staphylococcus* strains.

Species	Strain Type	Strain Identification Number ^1^	Resistant Antibiotics ^2^	MIC (µg·mL^−1^)		
				ZO	THO	TZ
*Escherichia coli*	Type	ATCC 25922	None	>500	500	>500
	MDR	ATCC BAA-2452	AMI, AMP, AZT, CEF, CTX, ETP, GEN, IMP, MER, PIP, TIC, TOB	>500	>500	>500
		ATCC BAA-2469	AMI, AMP, AZT, CEF, CIP, CTX, ETP, GEN, IMP, MER, NAL, NOR, PIP, TIC, TOB	>500	>500	>500
		ATCC BAA-2471	AMP, AZT, CEF, CIP, CTX, ETP, GEN, IMP, MER, NAL, NOR, PIP, TIC, TOB	>500	>500	>500
*Staphylococcus aureus*	Type	ATCC 25923	None	>500	>500	62.5
	MDR ^3^	MRSA1	MET, OXA	>500	>500	250
		MRSA2	MET, OXA	>500	>500	500
		MRSA3	MET, OXA	>500	>500	125
		MRSA4	MET, OXA	>500	>500	>500
*S. epidermidis*	Type	ATCC 14990	None	>500	>500	15.6
		KCTC 13171	None	>500	>500	31.3
	MDR ^4^	ATCC 12228	STR, AMP, PEN	>500	>500	15.6
*S. warneri*	Type	ATCC 27836	None	>500	>500	250

^1^ ATCC and KCTC represent American Type Culture Collection (https://www.atcc.org/; accessed on 17 June 2022) and Korean Collection for Type Cultures (https://kctc.kribb.re.kr/; accessed on 17 June 2022), respectively. ^2^ Acronyms: AMI, Amikacin; AMP, Ampicillin; AZT, Aztreonam; CEF, Cefepime; CTX, Cefotaxime; CIP, Ciprofloxacin; ETP, Ertapenem; GEN, Gentamicin; IMP, Imipenem; MER, Meropenem; MET, Methicillin; NAL, Nalidixic acid; NOR, Norfloxacin; OXA, Oxacillin; STR, Streptomycin; PEN, Penicillin; PIP, Piperacillin; TIC, Ticarcillin; TOB, Tobramycin. ^3^ The strain information is provided in a previous report [34]. ^4^ ATCC 12228 has been studied to understand the multiple antibiotic resistant potential mediated by efflux pump [35] and antibiotic exposures [36].

**Table 2:** Minimum inhibitory concentration (MIC) of antibiotics owing to TZ NC against the type *S. epidermidis* strain.

Antibiotics	Acronym	Subclass	MIC (μg·mL^−1^) ^1^	
			−TZ	+TZ
Ampicillin	AMP	β-lactam	0.125	0.0625
Chloramphenicol	CHL	Amphenicol	>4	4
Ciprofloxacin	CIP	Fluoroquinolone	<0.25	<0.25
Clindamycin	CLI	Lincosamide	<0.125	<0.125
Daptomycin	DAP	Cyclic lipopeptide	>1	1
Erythromycin	ERY	Macrolide	0.5	0.25
Gentamicin	GEN	Aminoglycoside	<0.5	<0.5
Levofloxacin	LEVO	Fluoroquinolone	0.25	0.125
Linezolid	LZD	Oxazolidinone	>2	1
Moxifloxacin	MXF	Fluoroquinolone	0.125	0.125
Nitrofurantoin	NIT	Nitrofuran	16	<8
Oxacillin +2%NaCl	OXA+	β-lactam	0.125	0.125
Penicillin	PEN	β-lactam	0.25	0.0625
Quinupristin/ Dalfopristin	SYN	Lincosamide	<0.125	<0.125
Rifampin	RIF	Rifampicin	<0.125	<0.125
Streptomycin	STR	Aminoglycoside	<250	<250
Tetracycline	TET	Tetracycline	>4	>4
Tigecycline	TGC	Tetracycline	0.125	0.03
Trimethoprim/sulfamethoxazole	SXT	Co-trimoxazole	0.25/4.75	0.5/9.5
Vancomycin	VAN	Glycopeptide	2	2

^1^ MIC values of the antibiotics were determined using Sensititre^TM^ Gram Positive MIC plate (Cat. No. GPALL1F, Thermo Fisher Scientific, Waltham, MA, USA) with a total volume of 200 μL. Three independent experiments were performed with ATCC 14990 cells using the 96-well platform as shown in Figure S7. Antibiotics with enhanced antibacterial activity owing to TZ NC are indicated in bold.

## Data Availability

Not applicable.

## Supplementary Materials

The following supporting information can be downloaded at: https://www.mdpi.com/article/10.3390/ijms23126770/s1.
