# Supplementary material for: Bioconjugated Thymol-Zinc Oxide Nanocomposite as a Selective and Biocompatible Antibacterial Agent against Staphylococcus Species
Source: Int J Mol Sci. 2022 Jun 17;23(12):6770. doi: 10.3390/ijms23126770 (PMC9224476; doi:10.3390/ijms23126770)
Supplement: Supplementary file 1 [file ijms-23-06770-s001.zip › ijms-1747635-supplementary.pdf]

Strain	MIC (μg·mL^{-1})		
	$n = 1$	$n = 2$	$n = 3$
	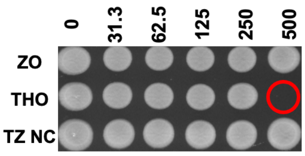	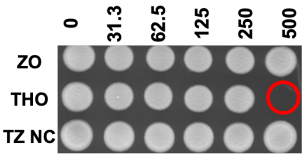	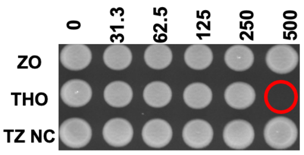
	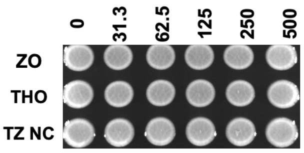	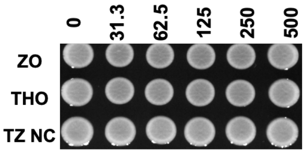	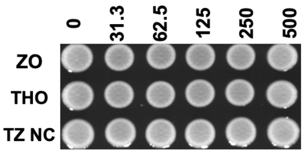
	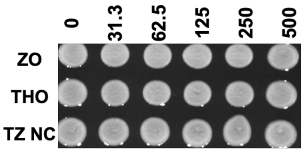	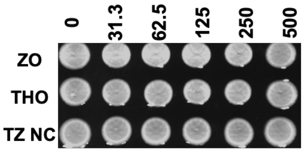	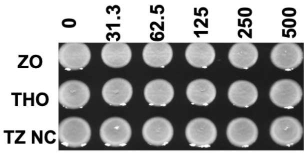
	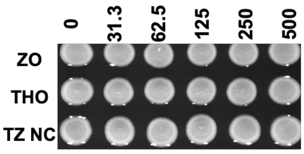	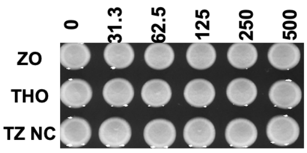	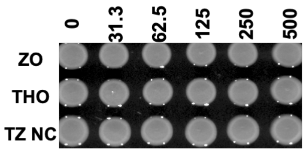
	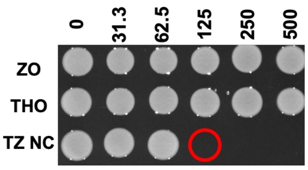	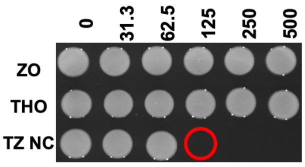	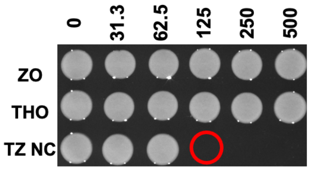
	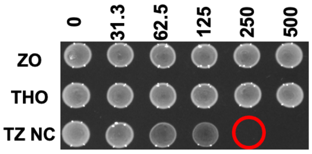	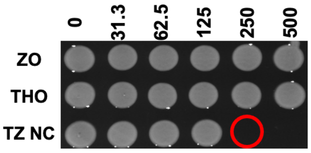	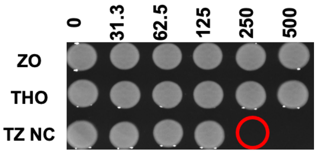
	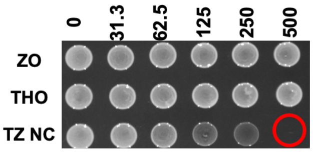	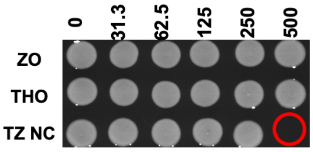	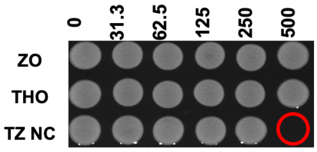

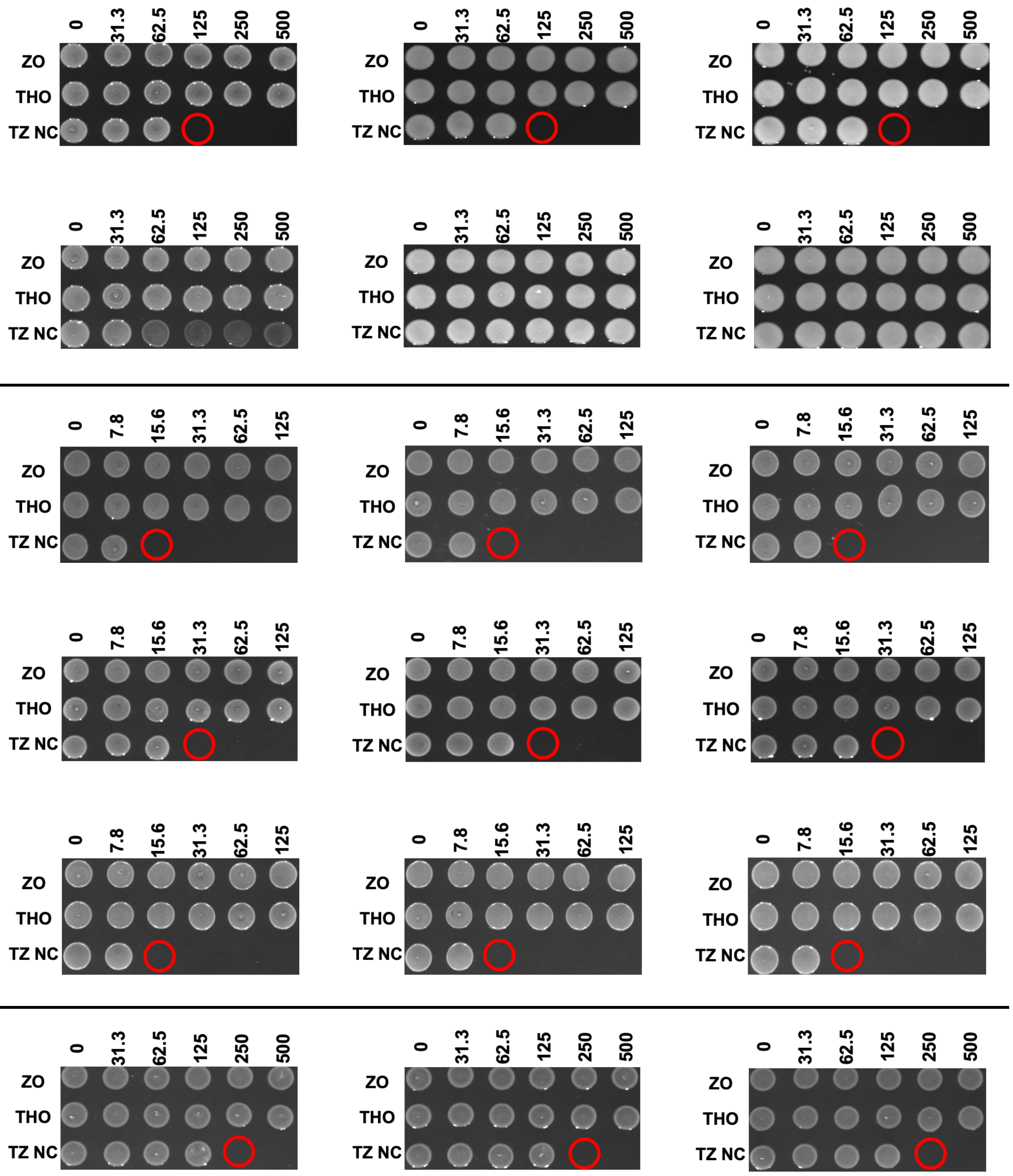

Figure S1. Cell viability assay. A fraction of cells treated with or without different concentrations of ZO, THO, and TZ NC against gram-negative *E. coli* or gram-positive *Staphylococcus* strains were spotted on LB agar plates and incubated at 37 °C for 24 h. The plate images were captured using ChemiDoc™ MP (Bio-Rad, Hercules, CA, USA) and ImageLab™ Software (ver.5.2.1, Bio-Rad, Hercules, CA, USA). All data of n = 3 are presented. MIC values, which are indicated by red circles, were the same as those listed in **Table 1**.

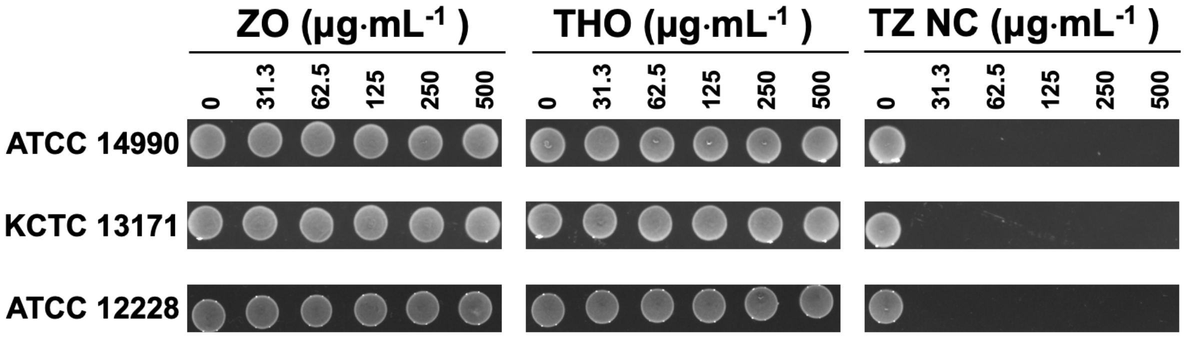

Figure S2. Cell viability assays. A fraction of cells treated with or without different concentrations (from 0 to 500 µg·mL^{-1}) of ZO, THO, and TZ NC against the *S. epidermidis* strains (ATCC 14990, KCTC 13171, and ATCC 14990) were spotted on LB agar plates and incubated at 37 °C for 24 h. The plate images were captured using ChemiDoc™ MP (Bio-Rad, Hercules, CA, USA) and ImageLab™ Software (ver.5.2.1, Bio-Rad, Hercules, CA, USA). One of the representative data from n = 3 is presented.

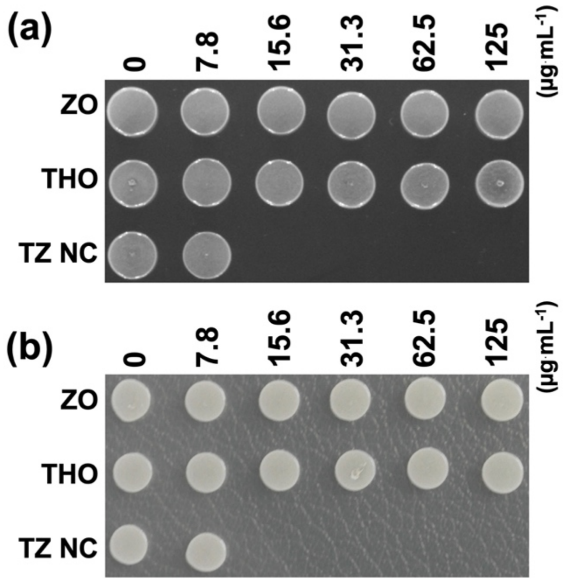

Figure S3. Cell viability assays. A fraction of cells treated with or without different concentrations (from 0 to 125 µg·mL^{-1}) of ZO, THO, and TZ NC against MDR *S. epidermidis* ATCC 12228 strain were spotted on LB agar plates and incubated at 37 °C for 24 h. The plate images were captured using (**a**) ChemiDoc™ MP (Bio-Rad, CA, USA) and ImageLab™ Software (ver.5.2.1, Bio-Rad, Hercules, CA, USA) or (**b**) digital camera (Samsung NX200, Suwon, Korea), respectively. One of the representative data from n = 3 is presented.

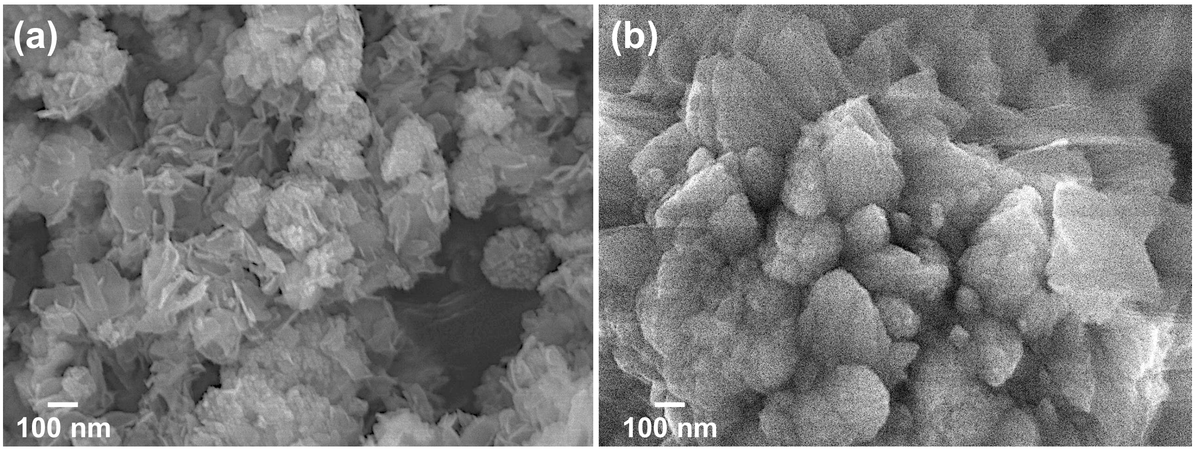

Figure S4. Field Emission Scanning electron microscopy (FE-SEM) images of (**a**) ZO and (**b**) TZ NC. The crystallite sizes of ZO and TZ NC were approximately 20 nm as confirmed by XRD analysis, and FE-SEM images of the two nanomaterials revealed that nanometer scale particles were densely aggregated on the surface to form a single large mass.

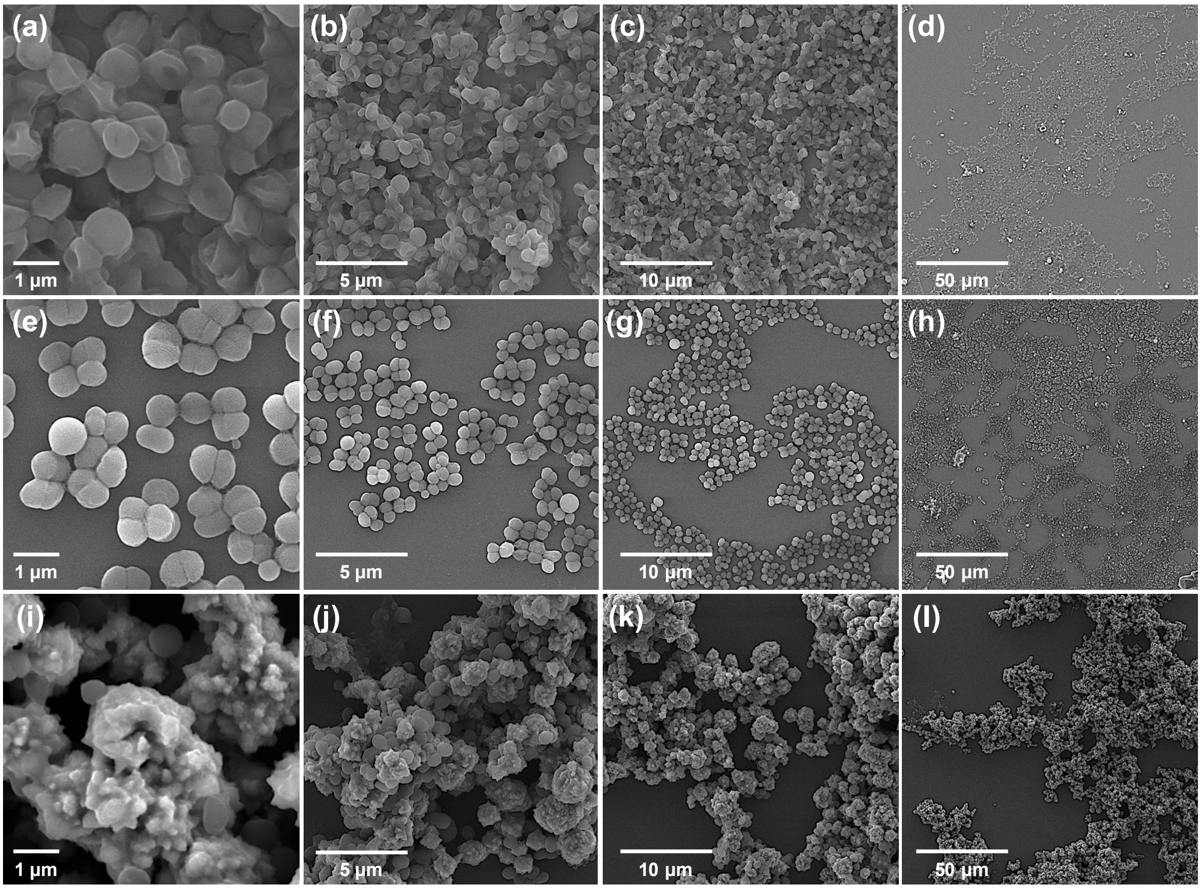

Figure S5. Cell morphology analysis. Scanning electron microscopy (SEM) images of type *S. aureus* strain ATCC 25923 treated without (**a**-**d**) or with either 1/4 MIC (**e**-**h**) or 1/2 MIC (**i**-**l**) of TZ NC, respectively. No membrane disruption was shown in 1/4 MIC of TZ NC treated cells (**e**-**h**) compared to non-treated cells. The whole membrane was aggregated with TZ NC in cells treated with 1/2 MIC of TZ NC (**i**-**l**), inducing partial membrane rupture.

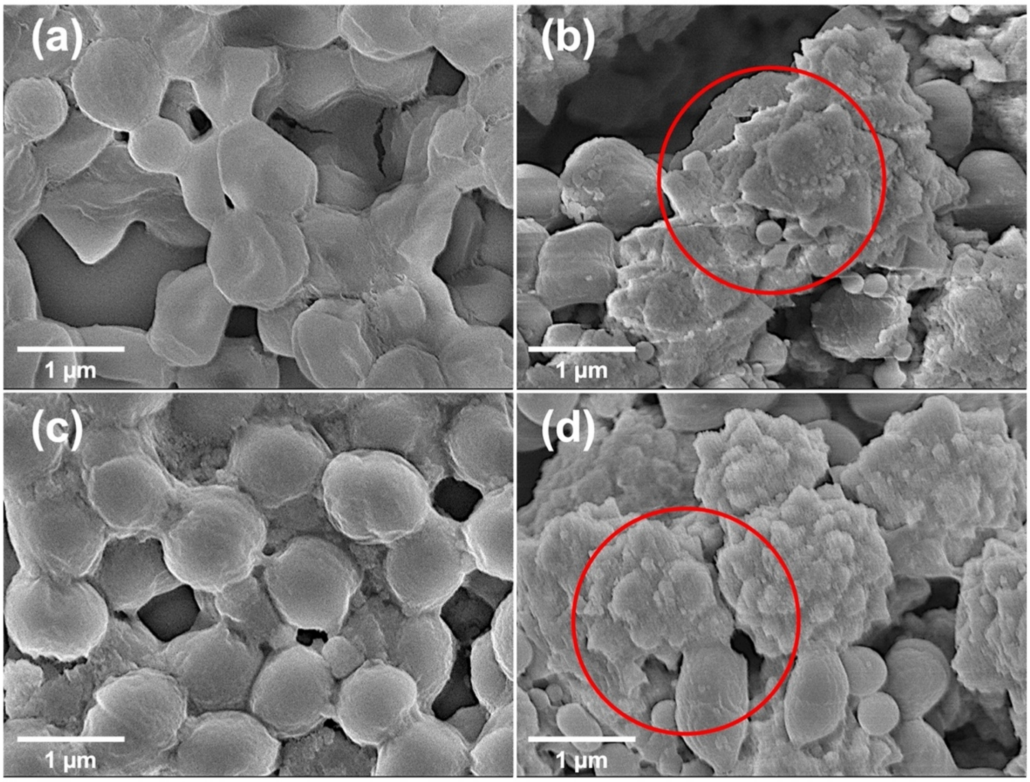

Figure S6. Cell morphology analysis. FE-SEM images of cells from type *S. aureus* ATCC 25923 (**a** and **b**) and MDR *S. epidermidis* ATCC 12228 (**c** and **d**) strains treated without (**a** and **c**) or with 1/2 MIC (**b** and **d**) of TZ NC, respectively. Membrane disruption with biofilm was not detected in non-TZ NC treated cells (**a** and **c**). However, whole membrane was aggregated with TZ NC in cells treated with 1/2 MIC of TZ NC (**b** and **d**, indicated in red circle), inducing partial membrane rupture.

(a)

µg·mL^{-1}	1	2	3	4	5	6	7	8	9	10	11	12
A	CHL				ERY					CLI		
	0.5	1	2	4	0.0625	0.125	0.25	0.5	1	0.125	0.25	0.5
b	DAP				OXA+					STR	DT1	DT2
	0.125	0.25	0.5	1	0.0625	0.125	0.25	0.5	1	250		
c	GEN				AMP							FOXS
	0.5	1	2	4	0.03	0.0625	0.125	0.25	0.5	1	2	1.5
d	LZD				PEN							
	0.25	0.5	1	2	0.015	0.03	0.0625	0.125	0.25	0.5	1	2
e	RIF				VAN							
	0.125	0.25	0.5	1	0.0625	0.125	0.25	0.5	1	2	4	8
f	SXT				LEVO					CIP		POS
	0.125/2.375	0.25/4.75	0.5/9.5	1/19	0.0625	0.125	0.25	0.5	1	0.25	0.5	
g	SYN				TGC					NIT		POS
	0.125	0.25	0.5	1	0.0075	0.015	0.03	0.0625	0.125	8	16	
h	TET				MXF					GEN	NEG	POS
	0.5	1	2	4	0.0625	0.125	0.25	0.5	1	125		

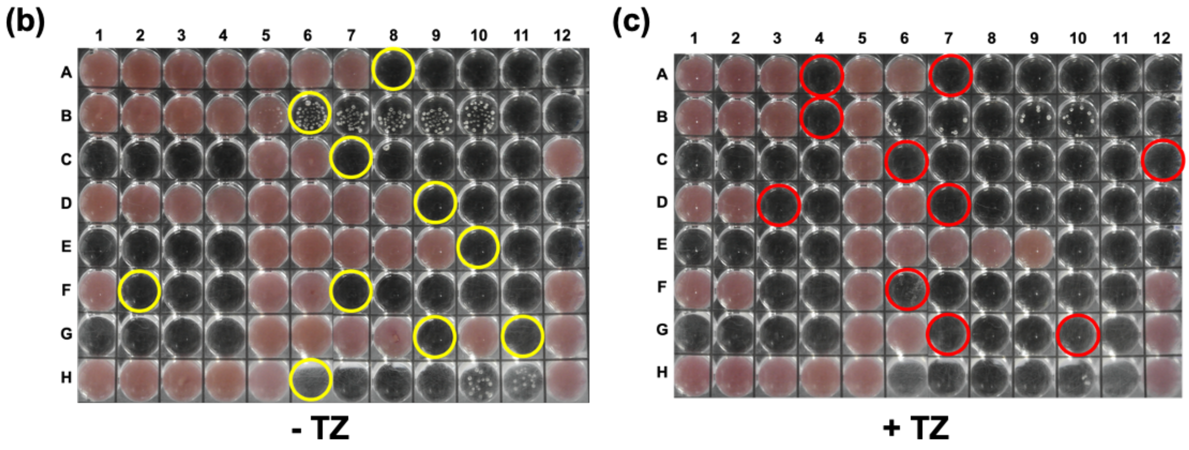

Figure S7. Screening of synergistic antibiotics (with TZ NC) against *S. epidermidis* ATCC 14990. (**a**) Information on antibiotics and the working concentration of individual antibiotics are shown. Acronym: AMP, Ampicillin; CHL, Chloramphenicol; CIP, Ciprofloxacin; CLI, Clindamycin; DAP, Daptomycin; ERY, Erythromycin; GEN, Gentamicin; LEVO, Levofloxacin; LZD, Linezolid; MXF, Moxifloxacin; NIT, Nitrofurantoin; OXA+, Oxacillin+2%NaCl; PEN, Penicillin; SYN, Quinupristin/Dalfopristin; RIF, Rifampin; STR, Streptomycin; TET, Tetracycline; TGC, Tigecycline; SXT, Trimethoprim/sulfamethoxazole; VAN, Vancomycin; DT1, D Test 1; DT2, D Test 2; FOXS, Cefoxitin; NEG and POS indicate the negative and positive controls, respectively. The antibiotics induced by TZ NC are indicated in red. Effect of the antibiotics on *S. epidermidis* (ATCC 14990) strain in the (**b**) absence or (**c**) presence of TZ NC (7.8 µg·mL^{-1}). Yellow or red circles on the plate indicate the MIC for individual antibiotics or synergistic antibiotics (with TZ NC), respectively. One of the representatives from n=3 is presented. All images were captured and processed using Adobe Illustrator CS6 (v16.0.0, Adobe Systems Inc., San Francisco, CA, USA). The MIC values for individual antibiotics are shown in **Table 2**.
